# The Influence of Vitamin D Receptor Gene Polymorphisms in Spondyloarthritis

**DOI:** 10.1155/2020/8880879

**Published:** 2020-12-08

**Authors:** Janisleya Silva Ferreira Neves, Jeane Eliete Laguila Visentainer, Denise Manjurma da Silva Reis, Marco Antonio Rocha Loures, Hugo Vicentin Alves, Fernanda Formaggi Lara-Armi, Josiane Bazzo de Alencar, Joana Maira Valentin Zacarias, Ana Maria Sell

**Affiliations:** ^1^Biosciences and Physiopathology, Department of Clinical Analysis and Biomedicine, Maringá State University, Paraná, Brazil; ^2^Department of Basic and Health Science, Maringá State University, Paraná, Brazil; ^3^Department of Medicine, Maringá State University, Paraná, Brazil

## Abstract

Spondyloarthritis (SpA) is an inflammatory rheumatic disease related to low bone mineral density. Because vitamin D plays an important role in bone metabolism and immune system modulation, the aim of this study was to evaluate the influence of polymorphisms in vitamin D receptor genes (*VDR*) in the development of SpA. In this case–control study, a total of 244 patients with SpA and 197 individuals with no SpA were included. Among the patients, 174 had ankylosing spondylitis (AS) and 66 had psoriatic arthritis (PsA). Genotyping of *Fok*I (rs2228570 C > T), *Bsm*I (rs1544410 C > T), *Apa*I (rs7975232 A > C), and *Taq*I (rs731236 T > C) was performed using PCR-RFLP, while genotyping of *HLA-B∗27* was performed using PCR-SSP. Serum levels for hydroxy (OH) vitamin D and the clinical activity index of the disease (BASDAI) were also evaluated. SNPStats and OpenEpi software were used for statistical analysis. The *Apa*I *a* allele and *Apa*I *a/a* genotype were less frequent in PsA compared with controls. The *Apa*I *a/a* genotype was associated with a protecting factor for PsA in females, and *Apa*I *A/a* was associated with a protecting factor for the disease in *HLA-B∗27* positive patients. Notwithstanding, the *Apa*I *a/a* genotype was a risk factor for SpA and AS in males. The *Fok*I *f/f* genotype was associated with a better clinical activity in PsA. When considering the covariates, vitamin D sufficiency, and gender, the *Fok*I *F/F* genotype was associated with a risk factor in males with SpA and AS compared with females with this same genotype. In conclusion, the *Apa*I rs7975232 polymorphism was associated with PsA, and the *Fok*I rs2228570 polymorphism was associated with better clinical PsA activity. *Apa*I and *Fok*I were associated with SpA and AS when considering gender and vitamin D sufficiency.

## 1. Introduction

Spondyloarthritis (SpA) is a term used for inflammatory diseases that are clinically, epidemiologically, and genetically related. They comprise a group of chronic rheumatic diseases classified as ankylosing spondylitis (AS), psoriatic arthritis (PsA), and reactive arthritis (ReA) and SpA which is related to inflammatory bowel disease (IBD-SpA) and undifferentiated SpA (uSpA) [1, 2]. The prevalence of SpA in the world ranges from 0.20 to 1.61% [1]. The most distinct form of the disease is AS, which is characterized by inflammation followed by progressive ankylosis of the sacroiliac joints and spine with peripheral arthritis and enthesopathy. PsA is an inflammatory arthritis associated with psoriasis which is manifested by peripheral arthritis, dactylitis, enthesitis, and spondylitis [3–6].

The important hereditability of SpA comes from HLA-B27 codified in the major histocompatibility complex (MHC) and defined in 1973 [7]. This association explains the 20% attributed to the heredity of the disease [2]. However, it does not completely explain the pathogenesis of the disease, when we consider that other genetic factors of susceptibility outside of the MHC have been uncovered. Most of them are defined through genome-wide association studies (GWAS) [8–11] and mainly include *ERAP1, IL23R*, and *RUNX3* [11].

The gradual clinical worsening in the manifestations of SpA is linked to inflammation and low bone mineral density of the joints, due to a high production of inflammatory cytokines [12] and vitamin D deficiency [13]. Vitamin D has a role in calcium homeostasis, bone metabolism, and immune system [14–16]. The biological effects of 1,25(OH)_2_D, an active form of vitamin D, are mediated by their receptor (vitamin D receptor (VDR)), a member of the nuclear hormone receptor superfamily expressed in almost all human cells including macrophages, lymphocytes, and dendritic cells. VDR complex influences the regulation of about 3% of the human genome [14]. It is a pleiotropic regulator of human physiology and modulates the immune system by suppressing the autoimmune process and tissue damage and delays chronic disease by inhibiting lymphocyte response via T helper 1 (Th1) and Th17 [17, 18]. Vitamin D deficiency may be associated with both susceptibility and severity of SpA [15, 19, 20].

The *VDR* gene is located on chromosome 12q13.11 and has 75 kb. The gene is composed of 11 exons, of which exons 2 and 3 encode the amino acids involved in DNA binding, and exons 7, 8 and 9 code amino acids involved in vitamin D binding [21, 22]. The commonly studied single nucleotide polymorphisms (SNPs) are *Fok*I C > T (rs2228570, where traditionally the *C* allele is designated as *F* and *T* allele as *f*, according to the RFLP restriction enzyme), *Apa*I A > C (rs7975232; traditionally, *A* allele is designated as *A* and *C* as *a*), *Taq*I C > T (rs731236; *C* allele is designated as *T* and the *T* allele as *t*), and *Bsm*I C > T (rs1544410; *C* allele is designated as *B* and T allele as *b*). The *Fok*I C > T change occurs in the first of the two potential translation initiation sites of exon 2, in the 5′ coding region of the *VDR*, and the ACG initiation codon results in a shorter peptide in the 3′ NH_2_ terminal (424 compared to 427), which has more efficient transcriptional activity. The *Bsm*I and *Apa*I (both in intron 8) and *Taq*I (in exon 9) polymorphisms are located near the regulatory 3′ untranslated region of the mRNA and do not determine changes in the structure of the receptor. They are in linkage disequilibrium (LD), and specific haplotypes have been shown to affect VDR mRNA stability and rate of transcription [23]. The influence of these polymorphisms on the development of many autoimmune and inflammatory diseases, as well as on psoriasis [29–33] and AS in Caucasians and Chinese [19, 34–36], has been documented [22, 24–28].

Because vitamin D is important in modulating the immune response and bone metabolism, the aim of this study was to evaluate a possible association between the polymorphisms in the vitamin D receptor gene (*VDR*), *Fok*I, *Bsm*I, *Apa*I, and *Taq*I, with SpA and its clinical forms. These polymorphisms were also correlated with the serum level of vitamin D and with the clinical activity index of the disease, Bath Ankylosing Spondylitis Disease Activity Index (BASDAI).

## 2. Materials and Methods

### 2.1. Sample Selection

The study was approved by the Research and Ethics Committee of the State University of Maringá (UEM), number CAAE 27723114. All subjects agreed to participate in the study and signed a consent form. A total of 244 patients with spondyloarthritis (SpA) were included in this study, and among them, 174 had ankylosing spondylitis (AS) and 66 had psoriatic arthritis (PsA). The diagnosis for SpA was performed through clinical, laboratory, and radiological criteria according to the ASAS (Assessment of Spondyloarthritis International Society) 2009/2011 criteria [37–39] by the rheumatologists from the University Regional Hospital of Maringá, and all patients had follow-ups with the same rheumatologists. Some patients had BASDAI (*N* = 141) and serum levels of hydroxyl (OH) vitamin D (*N* = 118) analyzed before starting treatment and none had vitamin D replacement at the time of diagnosis. The control group was selected by the same rheumatologists and was formed by 197 individuals with no SpA confirmed after clinical, laboratory, and radiological analyses and no vitamin D replacement (*N* = 23) at the time of the interview. The exclusion criteria for all samples were individuals with chronic diseases such as diabetes and other inflammatory and autoimmune diseases. Patients and controls were unrelated and matched by gender, age, and ethnicity. All participants were from the northwestern region of Paraná, in southern Brazil (22°29′30″-26°42′59″S and 48°02′24″-54°37′38″W), and were classified as mixed ethnicity, with predominantly European origin, based on the ethnic constitution in the state of Paraná as previously described [40] and confirmed for our region [41]. Based on this criterion, Asian descendants were excluded from the sample.

### 2.2. Technical Procedures

DNA was obtained from peripheral blood collected in vacuum tubes with EDTA using the salting-out method [42]. The concentration and quality of the DNA were analyzed using optical density in Nanodrop 2000® (ThermoScientific, Wilmington, USA).

The Polymerase Chain Reaction-Restriction Fragment Length Polymorphism (PCR-RFLP) technique was used for genotyping *VDR: Fok*I C > T (rs2228570) [43] and *Bsm*I C > T (rs1544410), *Apa*I A > C (rs7975232), and *Taq*I T > C (rs731236) [27]. This methodology was previously modified and validated for a population in our region [44].

Genotyping of *HLA-B∗27* was performed using Polymerase Chain Reaction-Sequence Specific Primers (PCR-SSP) according to sequences previously described [45]. This methodology was modified and validated in our population [46].

Serum levels of hydroxy (OH) vitamin D were determined using the chemiluminescence technique (Abbott Ireland Diagnostics Division, Lisnamuck, Longford, Ireland), following manufacturer's recommendations. The serum levels of vitamin D were classified as sufficient (>30 ng/mL) and insufficient (<29.9 ng/mL) [47].

The evaluation of the clinical disease activity index BASDAI was performed by the same rheumatologists according to criteria previously defined [48]. The evaluation covered all disease domains and consisted of six questions related to fatigue, spinal pain, pain and joint symptoms, pain due to entheses, and two other issues related to the quality and amount of pain associated with morning stiffness. The scores were defined from the visual analogical scale as 0 to 10 (0 = good; 10 = bad): scores < 3.99 indicated good clinical activity and >4.0 indicated high clinical activity.

### 2.3. Statistical Analysis

Student's *t*-test was used to calculate continuous data given as mean ± standard deviation (OpenEpi Version 3.01, https://www.openepi.com/Menu/OE_Menu.htm). The possible association of *VDR* polymorphisms with the disease was carried out using Chi-square and logistic regression, and for these analyses and for estimating the distribution of genotype frequencies according to the Hardy-Weinberg (HW) equilibrium, we applied the OpenEpi Version 3.01 and SNPStats software (https://www.snpstats.net/start.htm) [49]. The association tests were performed for codominant, dominant, recessive, overdominant, and log-additive genetic inheritance models and the better inheritance model was chosen using the lower Akaike information criterion (AIC) [49]. Gender, *HLA-B∗27*, BASDAI, and vitamin D serum levels were included as covariates in the analysis. Odds ratios and their respective 95% confidence intervals (CI) were defined only for statistically significant values, considering *P* value to be less than 5%. The Bonferroni adjustment for multiple testing was applied and the corrected value (*Pc*) for a truly significant value was obtained after the multiplication of *P* value by the number of analyzed SNPs: two SNPs when considering linkage disequilibrium between *Bsm*I, *Taq*I, and *Apa*I (*P* < 0.001; *Bsm*I-*Taq*I ∆′ = 0.516; *Bsm*I-*Apa*I ∆′ = 0.534; and *Apa*I-*Taq*I ∆′ = 0.657). The haplotype frequencies were estimated using the implementation of the expectation-maximization (EM) algorithm coded into the haplo.stats package. Bootstrapping by Haploview software version 4.2 was used to assess the robustness of the results [50]. QUANTO (http://www.biostats.usc.edu/software) was used to calculate the sample size using the less frequent allele (0.16 for *Apa*I), population risk of 1.5%, and OR = 2.0 for statistical power of 80%.

## 3. Results

The characteristics of SpA, AS, and PsA patients and controls are presented in Table 1. Patients and controls were matched by age and gender. The age of SpA patients was 48.58 (±15.50), and control, 40.70 (±12.30) years, which was similar for AS and PsA. Males represented 45.90% of the SpA patients and 41.11% of controls, which was similar for AS and PsA. *HLA-B∗27* was more frequent in SpA (35.66%), AS (44.47%), and PsA (15.15%) than in controls (6.59%), and these differences were statistically significant (*P* < 0.04; OR = 7.84, OR = 11.24, and OR = 2.53, resp.). The *HLA-B∗27:05* allele was also more frequent in SpA (16.39%), AS (20.40%), and PsA (6.81%) than in controls (2.28%) (*P* < 0.01; OR = 7.13, OR = 8.93, and OR = 3.13, resp.). BASDAI >4.0, which corresponds to severe clinical forms of the disease, was 72.91% in SpA, 75.52% in AS, and 66.66% in PsA. The serum level of the hydroxy (OH) vitamin D < 29.9 ng/mL (classified as insufficient) was 52.54% in SpA, 52.56% in AS, and 65.00% in PsA.

The correlation between serum levels of vitamin D, classified as sufficient (>30 ng/mL) and insufficient (<29.9 ng/mL), with the disease activity index (BASDAI <3.9 and >4.0), is shown in Table 2. Although worse clinical activity of the disease was observed in patients with vitamin D insufficiency, no statistical significance was found, which may be related to the low number of samples.

The genotype frequency distributions for all polymorphisms evaluated were in Hardy-Weinberg equilibrium (*P* > 0.05), which means they are good representatives of the control group and also ensure data quality. The genotype and allele frequency distributions of *VDR* were analyzed in patients and controls considering covariate genders (Tables 3–5) and gender plus vitamin D serum level (Table 6).

Significant differences were found in the *Apa*I genotype frequency distribution when PsA patients were compared with controls. The *Apa*I *a/a* genotype was less frequent in PsA when compared with controls in the codominant, dominant, and log-additive inheritance models, and the model of choice was the log-additive according to the minor AIC (*P*=0.011, *Pc* = 0.022; OR = 0.57, 95% CI = 0.37–0.89). In the log-additive inheritance model, each copy of *C* modifies the risk in an additive form; in other words, the homozygous *C/C*, or *Apa*I *a/a*, have a double risk of *T/C* or *Apa*I *A/a*. In addition, the *Apa*I *a* allele frequency was lower in patients with PsA than in controls (*P*=0.011, *Pc* = 0.022; OR = 0.60, 95% CI = 0.40–0.91). The genotype and allele frequency distributions of *Fok*I, *Bsm*I, and *Taq*I in the SpA, AS, and PsA patients were similar to those found in controls, when values were adjusted for gender covariates. These results are shown in Table 3.

After stratified analysis, and considering the distribution of *VDR* genotypes within and between genders, significant differences were also observed for *Apa*I (Table 4). The *Apa*I *a/a* genotype was associated with protection for PsA in females (*P*=0.001, *Pc* = 0.002; OR = 0.16). Otherwise, *Apa*I *a/a* genotype was associated with the risk of AS (*P*=0.018, *Pc* = 0.036; OR = 2.993) and SpA in males when this same genotype was compared with females (*P*=0.007, *Pc* = 0.014; OR = 3.58). These results are shown in Table 4.

When individuals with *HLA-B∗27* positive were separately analyzed, the *Apa*I *A/a* genotype was associated with protection for PsA, despite a small number of samples (Table 5).

In addition, despite the small number of patients with BASDAI and with serum levels of vitamin D determined at the time of diagnosis (for PsA, *N* = 40), the *Fok*I *f/f* genotype frequency was higher in the PsA patients with better clinical activity (BASDAI < 3.9; Table 5). The association was found in codominant and log-additive inheritance models. The latter was the model of choice according to the minor AIC, which means that each copy of *f* modifies the risk additively; that is, the homozygous *f/f* has double the risk compared with the *F/f* heterozygous.

When genotype and allele frequency distributions of *VDR* were analyzed in patients and controls when considering covariates vitamin D sufficiency and gender, *Fok*I and *Bsm*I variants were associated with the disease (Table 6). The *Bsm*I *b/b* genotype was more frequent in SpA than controls in dominant and log-additive inheritance models, but significance was lost after Bonferroni correction. The *Fok*I *F/F* genotype was more frequent in males with SpA, AS, and PsA than in females with this same genotype (OR = 5.81; OR = 5.74; OR = 6.67, resp.); however, significance was lost for PsA after Bonferroni correction. The large confidence interval is due to the small number of samples.

After the haplotype frequency distribution analysis, statistical significance was observed for the *Bsm*I_*Apa*I_*Taq*I *bAt* haplotype, which was a risk factor for PsA (haplotype frequency–hf = 0.078) compared with *baT* haplotype (hf = 0.328; *P*=0.038; OR = 2.24, 95% CI = 1.05–4.80). Perhaps, this was due to the influence of the *Apa*I polymorphism.

## 4. Discussion

Genetic polymorphisms have been studied in recent years in order to assist in the early diagnosis of spondyloarthritis and to understand the etiopathogenesis and improve treatment. Because vitamin D complex is important in the modulation of the immune response and in the homeostasis of calcium and phosphorus, the association between *VDR* polymorphisms and SpA, AS, and PsA was investigated. These associations were correlated with the covariates: gender, *HLA-B∗27*, BASDAI, and serum levels of vitamin D.

The main finding of this study was that the *ApaI* polymorphism was associated with psoriatic arthritis: *Apa*I *a/a* was a protective factor for PsA and for women with PsA, and *Apa*I *A/a* was a protective factor for PsA patients who were HLA*-B∗27* positive. Furthermore, the *Fok*I *f/f* genotype contributed to less clinical disease activity in PsA. For SpA and AS, the *Apa*I *a/a* and *Fok*I *F/F* genotypes were risk factors for male patients. To our knowledge, this is the first study to address the association of *VDR* polymorphism with PsA.

Psoriatic arthritis is a chronic inflammatory musculoskeletal disease associated with psoriasis. Vitamin D is involved in calcium homeostasis, bone formation, osteoclastogenesis, and osteoclast activity and in the regulation of immune response. The action of vitamin D depends on vitamin D receptor (VDR). Many studies have been conducted to determine the association between *VDR* polymorphisms and psoriasis, but few have included patients with PsA. In meta-analysis studies, *Apa*I, *Taq*I, *Bsm*I, and *Fok*I polymorphisms and susceptibility to psoriasis were evaluated in Caucasians and Asians [29, 31, 33]. These studies indicated an association between psoriasis and the *Taq*I *T/T* genotype in Caucasians [31, 33], but not in Asians [29]. According to the *ApaI* polymorphism, only one study found that *ApaI a/a*, in a dominant inheritance model, was a risk factor for psoriasis in Caucasians [29]; however, the *Apa*I *A* allele was a risk factor for psoriasis in Chinese [30]. We found that the *Apa*I variant was a protective factor for PsA, especially in women and *HLA-B∗27* positive patients. Our findings may be explained by autoimmunity and by altered bone remodelling in psoriatic arthritis. In other studies, *Apa*I *a/a* was found to be a protector for idiopathic scoliosis [51], and it was associated with higher levels of type I collagen propeptides [52]. In addition, the *Apa*I *a* allele correlated with higher levels of vitamin D in the serum [53]. Although we had a small number of patients with BASDAI and vitamin D serum levels determined at the time of diagnosis, the *Fok*I *f/f* genotype was a protective factor for better clinical activity in PsA. Previously, *Fok*I polymorphism was associated with lower bone mineral density [52] and *Fok*I *F/F* or *F/f* was a susceptibility factor for rheumatoid arthritis [25]. Due to the clinical heterogeneity of PsA, the identification of genetic susceptibility loci is important [9].

Ankylosing spondylitis is a chronic inflammatory disease involving the axial skeleton, regions of enthesis, and peripheral joints and predominantly affects males [13]. In this study, *VDR* polymorphisms were not associated with AS. However, in a stratified analysis considering gender, the *Apa*I *a/a* was a susceptibility factor for AS and SpA in men, and *Fok*I *F/F* was a risk factor for AS and SpA when compared with the same genotype in women. *Apa*I *a/a* was previously correlated with lower bone mineral density [54–56] and with vertebral osteoporosis in AS, contributing to spinal deformity and bone pain [34]. *Apa*I *a/a* was also associated to be a susceptibility factor for rheumatoid arthritis [57]. To better understand the influence of *Apa*I polymorphism in spondyloarthropathies, the correlation between different bone disorders and inflammatory processes in different clinical forms of the disease must be considered. Regarding the *Fok*I polymorphism, studies have shown that *Fok*I genotypes were associated with the lumbar but not the peripheral bone mineral density in male and Caucasian patients with AS, influencing inflammatory and pain indices [34, 35]. We found that the *Fok*I *F/F* was a risk factor for AS after stratification with vitamin D. This result could be compatible with the lower osteocalcin scores previously associated with AS patients with the *Fok*I *F/F* genotype [34]. Osteocalcin is an important osteoblastic product and its action is stimulated by vitamin D3 [58]. Other *VDR* polymorphisms associated with AS were found in two Chinese studies, and *Taq*I *G* allele and the *Taq*I haplotype for rs11168266 and rs11168267 were risk factors for the disease [36, 59].

Vitamin D deficiency has been associated with symptoms of SpA, such as pain, inflammation, and structural damage due to altered bone activity [15, 60–62]. Experimental studies show that vitamin D interferes in the molecular pathways of the disease, especially regarding entheseal inflammation and ossification involving IL-23 cytokines and sclerostin [15]. Because vitamin D deficiency is pandemic, we had difficulty in selecting patients without hormonal therapy and the number of SpA patients classified according to BASDAI and serum vitamin D determination was small. In our study, patients with vitamin D deficiency seem to present worse clinical activity (BASAI >4.0), but no statistical significance was found. In addition, despite the small number of patients, the *Bsm*I *b/b* genotype was associated with the risk of SpA, though significance was lost after Bonferroni correction. Other studies conducted with rheumatoid arthritis have shown that the *Bsm*I *b/b* genotype was a susceptibility risk factor for the disease [25, 57].

*HLA-B∗27* is the main genetic factor associated with SpA and is present in about 90% of patients with AS and 20% with PsA in nonmixed populations [10, 63]. *HLA-B∗27* has high polymorphisms named *HLA-B∗27:01* to *HLA-B∗27:226* (http://hla.alleles.org/alleles/class1.html). The most common subtypes associated with AS are *HLA-B∗27:*05, an ancestral allele from which other subtypes have derived (in Caucasians), *HLA-B∗27:04* (Chinese), and *HLA-B∗27:02* (Mediterranean populations). Two subtypes *HLA-B27∗06* and *HLA-B27∗09* were not associated with SpA [63–65]. In this study, *HLA-B∗27* and *HLA-B∗27:05* were associated with SpA (with respective frequencies being 35.66% and 16.39%), AS (44.25% and 20.40%), and PsA (15.15% and 6.81%) when compared with controls. For controls, the *HLA-B∗27* frequency was 6.59% and *HLA-B∗27:05* was 2.28%, according to what is expected for the Brazilian population (http://www.allelefrequencies.net/). The *HLA-B∗27* frequency is lower in SpA in populations with a higher degree of heterogeneity, such as in Latin America, and for AS it was found in 50% to 70% of Brazilian and Ibero-American patients [66–68].

The positive points of this study were the judicious selection of patients and controls, which had the diagnostics confirmed by an experienced rheumatologist. In addition, because SpA was primarily linked to males but has been recognized as an important cause of disability among females [69], the patients and controls were matched by gender. Considering the importance of age in the criteria for classification of spondyloarthritis [70], patients and controls were matched by age. The main limitation of our study was the low statistical power of analysis when some subgroups were analyzed. Another point is that it was not possible to correlate *VDR* polymorphisms with serum levels of vitamin D before initiating treatment protocol in all patients. Studies conducted with patients before initiating treatment protocol and with follow-ups for their clinical evolution may contribute to a better understanding of the pathogenesis of the vitamin D complex in the SpA.

## 5. Conclusion

In conclusion, *VDR* polymorphisms were associated with psoriatic arthritis: *ApaI* (7975232) polymorphism was a protective factor for disease and *Fok*I (rs2228570) polymorphism was associated with better clinical disease activity. *Apa*I and *Fok*I were associated with SpA and AS only when gender and vitamin D sufficiency was considered.

## Figures and Tables

**Table 1:** Characteristics of patients with spondyloarthritis (SpA), ankylosing spondylitis (AS), and psoriatic arthritis (PsA) and controls.

Variable	SpA *N* = 244	AS *N* = 174	PsA *N* = 66	Controls *N* = 197	OR (95% CI)	*P* value
Age mean ± SD (year)	48.58 (±15.50)	46.37 (±15.16)	54.79 (±14.68)	40.70 (±12.30)		
Gender male, *n* (%)	112 (45.90)	77 (44.25)	27 (40.90)	81 (41.11)		
*HLA-B* ^*∗*^ *27, n* (%)	87 (35.66)^a^	77 (44.47)^b^	10 (15.15)^c^	13 (6.59)	7.84 (4.22–14.59)^a^ 10.96 (5.39–22.31)^b^ 2.53 (1.05–6.08)^c^	<0.0001 <0.0001 0.04
*HLA-B* ^*∗*^ *27:02, n* (af)	5 (0.010)	4 (0.011)	1 (0.008)	0 (0)		
*HLA-B* ^*∗*^ *27:05, n* (af)	80 (0.164)^a^	71 (0.204)^b^	9 (0.068)^c^	9 (0.023)	7.13 (3.53–14.39)^a^ 8.93 (4.60–17.33)^b^ 3.13 (1.22–8.06)^c^	<0.0001 <0.0001 0.013
*HLA-B* ^*∗*^ *27:08, n* (af)	1 (0.002)	1 (0.003)	0 (0)	1 (0.002)		
*HLA-B* ^*∗*^ *27:09, n* (af)	0 (0)	0 (0)	0 (0)	1 (0.002)		
*HLA-B* ^*∗*^ *27:12, n* (af)	0 (0)	0 (0)	0 (0)	1 (0.002)		
BASDAI, *n* (%)	*N* = 203	*N* = 143	*N* = 60			
<3.9	55 (27.09)	35 (24.48)	20 (33.33)			
>4.0	148 (72.91)	108 (75.52)	40 (66.66)			
Vitamin D, *n* (%)	*N* = 118	*N* = 78	*N* = 40			
<29.9 ng/mL	62 (52.54)	41 (52.56)	26 (65.00)			
>30.0 ng/mL	56 (47.46)	37 (47.44)	14 (35.00)			

*N* = total number of individuals; *n* = number of individuals; af = allele frequency; SD = standard deviation; OR = odds ratio; CI = confidence interval; *P* value <0.05 was statistically significant. BASDAI: Bath Ankylosing Spondylitis Disease Activity Index. Vitamin D: serum levels of hydroxy (OH) vitamin D. ^a^SpA *vs.* controls; ^b^AS *vs.* controls; ^c^PsA vs. controls.

**Table 2:** Comparison between vitamin D serum levels and BASDAI in the spondyloarthritis (SpA), ankylosing spondylitis (AS), and psoriatic arthritis (PsA) patients.

	SpA *N* = 118		AS *N* = 78		PsA *N* = 40	
BASDAI	<3.9	>4.0	<3.9	>4.0	<3.9	>4.0
Vitamin D	*n* (%)	*n* (%)	*n* (%)	*n* (%)	*n* (%)	*n* (%)
>30 ng/mL	15 (12.7)	34 (29.7)	9 (11.5)	26 (33.3)	6 (15.0)	8 (20.0)
<29.9 ng/mL	12 (10.2)	50 (42.4)	5 (6.4)	31 (39.7)	7 (17.5)	19 (47.5)

*N* = total number of individuals; *n* = number of individuals; BASDAI: Bath Ankylosing Spondylitis Disease Activity Index; vitamin D: serum levels of hydroxyl (OH) vitamin D. No statistical significance was observed.

**Table 3:** Genotype and allele frequency distributions of *Fok*I, *Bsm*I, *Taq*I, and *Apa*I in patients with spondyloarthritis (SpA), ankylosing spondylitis (AS), and psoriatic arthritis (PsA) and controls.

Genotypes alleles	SpA *N* = 244 *n* (*f*)	AS *N* = 174 *n* (*f*)	PsA *N* = 66 *n* (*f*)	Controls *N* = 197 *n* (*f*)	*P*	*Pc*	OR (95% CI)
*Fok*I							
*F/F*	103 (0.422)	80 (0.460)	23 (0.349)	86 (0.436)			
*F/f*	116 (0.475)	75 (0.431)	38 (0.576)	94 (0.477)			
*f/f*	25 (0.102)	19 (0.109)	5 (0.760)	17 (0.086)			
*F*	322 (0.659)	235 (0.675)	84 (63.6)	266 (0.675)			
*f*	166 (0.340)	113 (0.325)	48 (36.4)	128 (0.325)			
*Bsm*I							
*b/b*	85 (0.348)	60 (0.345)	22 (0.333)	76 (0.386)			
*B/b*	128 (0.525)	93 (0.535)	35 (0.530)	96 (0.487)			
*B/B*	31 (0.127)	21 (0.121)	9 (0.137)	25 (0.127)			
*b*	298 (0.611)	213 (0.612)	79 (0.598)	248 (0.629)			
*B*	190 (0.389)	135 (0.388)	53 (0.402)	146 (0.371)			
*Apa*I							
*A/A*	80 (0.328)	51 (0.293)	29 (0.439)	58 (0.294)			
*A/a*	118 (0.484)	85 (0.489)	32 (0.485)	106 (0.538)			
*a/a*	46 (0.189)	38 (0.218)	5 (0.076)^a^	33 (0.168)	0.011	0.022	0.57 (0.37–0.89)^a^
*A*	278 (0.570)	187 (0.537)	90 (0.682)	222 (0.563)			
*a*	210 (0.430)	161 (0.463)	42 (0.318)^a^	172 (0.437)	0.011	0.022	0.60 (0.40–0.91)^a^
*Taq*I							
*T/T*	101 (0.414)	72 (0.414)	26 (0.394)	81 (0.411)			
*T/t*	114 (0.467)	83 (0.477)	31 (0.470)	95 (0.482)			
*t/t*	29 (0.119)	19 (0.109)	9 (0.136)	21 (0.107)			
*T*	316 (0.648)	227 (0.652)	83 (0.629)	257 (0.652)			
*t*	172 (0.352)	121 (0.348)	49 (0.371)	137 (0.348)			

*N* = total number; *n* = number of individuals with the allele or genotype; *f* = allele or genotype frequency; *Pc* = Bonferroni adjustment for multiple testing; OR = odds ratio; CI = confidence interval. Values were adjusted for gender. ^a^PsA *vs*. controls: *Apa*I *a/a*, log-additive inheritance model.

**Table 4:** Association between *Apa*I and spondyloarthritis (SpA), ankylosing spondylitis (AS), and psoriatic arthritis (PsA) within and between genders.

		SpA^a^ (*N* = 244)	AS^b^ (*N* = 174)	PsA^c^ (*N* = 66)	Controls (*N* = 197)	*P* value	*Pc*	OR (95% CI)
*Apa*I								
Female	*A/A*	48 (0.364)	31 (0.333)	17 (0.436)	32 (0.276)			Ref.
	*A/a*	66 (0.500)	47 (0.505)	20 (0.513)	61 (0.526)			
	*a/a*	18 (0.136)	15 (0.161)	2 (0.051)^c^	23 (0.1980	0.001	0.002	0.16 (0.02–0.47)^c^
Male	*A/A*	32 (0.286)	20 (0.247)	12 (0.444)	26 (0.321)			Ref.
	*A/a*	52 (0.464)	38 (0.469)	12 (0.444)	45 (0.556)			
	*a/a*	28 (0.250)	23 (0.284)^b^	3 (0.111)	10 (0.123)	0.018	0.036	2.99 (1.16–7.68)^b^

*Apa*I								
Female Male	*A/A* *A/A*	48 (0.60) 32 (0.40)			32 (0.552) 26 (0.448)			
Female Male	*A/a* *A/a*	66 (0.559) 52 (0.441)			61 (0.575) 45 (0.425)			
Female Male	*a/a* *a/a*	18 (0.391) 28 (0.609)^a^			23 (0.697) 10 (0.303)			Ref.
						0.007	0.014	3.58 (1.38–9.25)^a^

Only significant data are shown. *N* = total number; OR = odds ratio; CI = confidence interval; Ref = reference; *Pc* = Bonferroni adjustment for multiple testing. ^a^SpA *vs*. control; ^b^AS *vs*. control; ^c^PsA *vs*. control.

**Table 5:** Association between *Apa*I and *Fok*I genotypes with psoriatic arthritis (PsA) classified according to the *HLA-B∗27* presence and the BASDAI.

*HLA-B* ^*∗*^ *27* presence^*∗*^						
		PsA (*N* = 13)	Control (*N* = 10)	*P* value	*Pc*	OR (95% CI)
*Apa*I	*AA*	1 (0.077)	6 (0.600)	Ref.		
	*Aa*	10 (0.769)	4 (0.400)	0.02	0.04	0.07 (0.01–0.75)
	*aa*	2 (0.154)	0 (0.000)			

BASDAI^*∗∗*^ PsA (*N* = 40)						
		BASDAI < 3.9	BASDAI > 4.0	*P* value	*Pc*	OR (95% CI)
*Fok*I	*FF*	2 (0.154)	12 (0.444)	Ref.		
	*Ff*	8 (0.615)	14 (0.519)			
	*ff*	3 (0.231)	1 (0.037)	0.02	0.04	0.25 (0.07–0.91)

All genotypes *Fok*I, *Taq*I, *Apa*I, and *Bsm*I were analyzed, but only significant results were shown. *Pc* = Bonferroni adjustment for multiple testing; *N* = total number; OR = odds ratio; CI = confidence interval; Ref = reference; BASDAI: Bath Ankylosing Spondylitis Disease Activity Index. ^*∗*^Only *HLA-B*^*∗*^*27* positive individuals were analyzed. ^*∗∗*^PsA patients were analyzed, and significance was found in the log-additive inheritance model.

**Table 6:** Association between *Fok*I and *Bsm*I with spondyloarthritis (SpA), ankylosing spondylitis (AS), and psoriatic arthritis (PsA) considering covariates gender and vitamin D.

		SpA^a^ (*N* = 118)	AS^b^ (*N* = 78)	PsA^c^ (*N* = 40)	Controls (*N* = 23)	*P* value	*Pc*	OR (95% CI)
*BsmI*								
*All*	*B/b*	40 (0.339)			13 (0.565)			
	*B/b*	66 (0.559)			9 (0.391)			
	*b/b*	12 (0.102)^a^			1 (0.043)	0.036	0.072	2.36 (1.02–5.46)^*∗*^

*FokI*								
Female	*F/F*	22 (0.431)	16 (0.432)	6 (0.429)	10 (0.833)			Ref.
						0.01 0.01	0.02 0.02	5.81 (1.11–30.29)^a^ 5.74 (1.08–30.54)^b^
Male	*F/F*	29 (0.569)^a^	21 (0.595)^b^	8 (0.571)^c^	2 (0.167)	0.04	0.08	6.67 (1.05–42.43)^c^

Only significant data are shown. *N* = total number; OR = odds ratio; CI = confidence interval; Ref = reference; *Pc* = Bonferroni adjustment for multiple testing. *∗*Log-additive inheritance model. ^a^SpA *vs*. control; ^b^AS *vs*. control; ^c^PsA *vs*. control.

## Data Availability

The genotype and alelle data used to support the findings of this study are included within the article.
